# Ethnicity and cardiovascular health inequalities in people with severe mental illnesses: protocol for the E-CHASM study

**DOI:** 10.1007/s00127-016-1185-8

**Published:** 2016-02-04

**Authors:** J. Das-Munshi, M. Ashworth, F. Gaughran, S. Hull, C. Morgan, J. Nazroo, A. Roberts, D. Rose, P. Schofield, R. Stewart, G. Thornicroft, M. J. Prince

**Affiliations:** Department of Health Service and Population Research, Centre for Epidemiology and Public Health, Institute of Psychiatry, Psychology and Neuroscience, King’s College London, De Crespigny Park, PO 33, London, SE5 8AF UK; Division of Health and Social Care Research, Department of Primary Care and Public Health Sciences, King’s College London, 3rd Floor, Addison House, Guy’s Campus, London, SE1 1UL UK; South London and Maudsley Trust and King’s College London, London, UK; Centre for Primary Care and Public Health, Blizard Institute, Queen Mary University of London, Yvonne Carter Building, 58 Turner Street, London, E1 2AB UK; University of Manchester, Manchester, England; Natural Language Processing Group, Department of Computer Science, University of Sheffield, Sheffield, England; Institute of Psychiatry, Psychology and Neuroscience, King’s College London, Room M1.06, De Crespigny Park, London, SE5 8AF UK

**Keywords:** Severe mental illness, Ethnicity, Cardiovascular disease, Schizophrenia, Bipolar affective disorder

## Abstract

**Purpose:**

People with severe mental illnesses (SMI) experience a 17- to 20-year reduction in life expectancy. One-third of deaths are due to cardiovascular disease. This study will establish the relationship of SMI with cardiovascular disease in ethnic minority groups (Indian, Pakistani, Bangladeshi, black Caribbean, black African and Irish), in the UK.

**Methods:**

E-CHASM is a mixed methods study utilising data from 1.25 million electronic patient records. Secondary analysis of routine patient records will establish if differences in cause-specific mortality, cardiovascular disease prevalence and disparities in accessing healthcare for ethnic minority people living with SMI exist. A nested qualitative study will be used to assess barriers to accessing healthcare, both from the perspectives of service users and providers.

**Results:**

In primary care, 993,116 individuals, aged 18+, provided data from 186/189 (98 %) practices in four inner-city boroughs (local government areas) in London. Prevalence of SMI according to primary care records, ranged from 1.3–1.7 %, across boroughs. The primary care sample included Bangladeshi [*n* = 94,643 (10 %)], Indian [*n* = 6086 (6 %)], Pakistani [*n* = 35,596 (4 %)], black Caribbean [*n* = 45,013 (5 %)], black African [*n* = 75,454 (8 %)] and Irish people [*n* = 13,745 (1 %)]. In the secondary care database, 12,432 individuals with SMI over 2007–2013 contributed information; prevalent diagnoses were schizophrenia [*n* = 6805 (55 %)], schizoaffective disorders [*n* = 1438 (12 %)] and bipolar affective disorder [*n* = 4112 (33 %)]. Largest ethnic minority groups in this sample were black Caribbean [1432 (12 %)] and black African (1393 (11 %)).

**Conclusions:**

There is a dearth of research examining cardiovascular disease in minority ethnic groups with severe mental illnesses. The E-CHASM study will address this knowledge gap.

## Background

People living with severe mental illnesses such as schizophrenia have a reduced life expectancy relative to the general population which is up to 20 years earlier in men and 17 years in women, in high income countries [1, 2]. A large proportion of deaths are from chronic diseases, including coronary heart disease and stroke [3]. Increased mortality may be related to people with severe mental illnesses receiving poorer quality physical healthcare [4–6]. There is also a higher prevalence of metabolic risk factors such as obesity [7], hyperlipidaemia [8], diabetes [9] together with higher smoking rates [10], in these populations. Anti-psychotic medications, especially at higher doses, are associated with death from stroke and coronary heart disease [11]. People with severe mental illnesses experience barriers to seeking timely help for co-morbid medical problems [12]. Finally, there may be shared factors underlying premature mortality and severe mental illness, such as social disadvantage [12].

Health inequalities may be even more pronounced among ethnic minority populations with severe mental illnesses [13, 14]. The reasons for this are unclear. There is much evidence to suggest that those from ethnic minority groups may experience disadvantage within the mental healthcare system. Black people are more likely to be compulsorily detained [15], be less satisfied with prescribing [16] and more likely to be prescribed high-potency antipsychotics at high doses [17–19]. In addition, physical health monitoring may not be to the same standard as for white patients [20]. The prevalence of cardiovascular disease is known to be elevated in people living with severe mental illnesses and is also known to be of a greater concern for some ethnic minority groups [21]. The exact nature of the interaction between being of an ethnic minority background and living with severe mental illness, for the risk of cardiovascular disease—is less clear. Previous research examining cardiovascular disease health inequalities by ethnicity in severe mental illness populations has been limited by an over-reliance on small convenience samples recruited from clinics, with limited representativeness and without the inclusion of adequate numbers of people from ethnic minority groups to enable assessment of prevalence of cardiovascular disease [14].

The E-CHASM study (ethnicity and cardiovascular health inequalities in severe mental illness) described in this protocol will draw upon electronic health records from a large secondary care mental health Trust in England, as well as data from primary care, to enable analyses examining the mechanisms for premature mortality due to cardiovascular disease in ethnic minority people with severe mental illnesses. The catchment areas for the study (south east/east London) represent an ethnically and socioeconomically diverse part of London, typical of many inner-cities where ethnic minority communities reside and where the burden of chronic health conditions is greatest.

E-CHASM will additionally utilise an embedded qualitative study, with integration of qualitative findings with quantitative findings, adopting a mixed methods design [22]. Using this approach, it may be possible to understand trends revealed in quantitative data analysis, particularly from the perspectives of service users, carers and clinicians/service providers, which will help to elucidate mechanisms underlying quantitative findings.

## Objectives

- To understand the reasons for premature mortality in ethnic minority people living with severe mental illnesses; in particular to determine variations by ethnicity in the following:
- The effect of severe mental illness on cardiovascular risk factors;
- Cause-specific mortality patterns among people with severe mental illnesses;
- Quality of care received, relevant to premature mortality;
- To develop and validate a measure for individual-level socioeconomic position for application in a large secondary care electronic mental health records data resource, which will be used to improve the assessment of the association of self-ascribed ethnicity with health-related outcomes.
- To assess barriers to equitable physical healthcare amongst ethnic minority people living with severe mental illnesses, from the perspectives of service users, their carers and clinicians.

## Hypotheses (for quantitative data analyses)

Compared to white British people with severe mental illnesses, ethnic minority service users with severe mental illnesses will:

1. Have an elevated prevalence of cardiovascular disease risk factors (hypertension, diabetes, obesity, raised serum cholesterol, and smoking) and be less likely to have had these adequately managed, as determined by national standards for clinical management.
2. Be less likely to have cardiovascular disease risk factors adequately screened or managed, when prescribed neuroleptic medication;
3. Be more likely to be prescribed multiple antipsychotics or antipsychotics at higher doses or outside recommended dose ranges.
4. Experience differing causes of mortality (i.e., cause-specific mortality fractions), in particular excess risk of mortality due to coronary artery disease.

## Qualitative study aims

The aim of the qualitative phase of this study is to identify barriers to delivering equitable physical healthcare (from clinician perspectives) or in accessing healthcare (service user and/or carer perspectives).

## Qualitative study questions

1. What are the barriers to having physical health monitored and treated in black and minority ethnic people living with severe mental illness?
2. How are physical health problems related to cardiovascular disease managed by black and minority ethnic participants living with severe mental illnesses?

## Methods

### Design

To address key objectives, this study will utilise anonymised patient data from a variety of sources, some of which will be linked together (e.g., secondary care patient data linked to Office for National Statistics (ONS) mortality data). This programme of research will follow a quantitative study design leading on to a nested qualitative study. In the final phase, results from the quantitative and qualitative studies will be integrated.

### Quantitative research methodology

#### Overview of data from primary care

##### Setting

The London boroughs represented in the study (Lambeth, Tower Hamlets, Newham, City and Hackney) are notable for being home to the largest ethnic minority communities in the UK, including Bangladeshi, black Caribbean and black African communities; up to 51 % in these areas comprise people of an ethnic minority background [23]. These areas are also characterised by high population density and poverty [23, 24]. Tower Hamlets, Newham and Hackney have the highest levels of deprivation in England [25]. The location of the study is characteristic of many other urban locations where ethnic minority communities reside within the UK [24].

*Measures from primary care* The quality and outcomes framework (QOF), a pay-for-performance scheme [26] introduced into primary care in the UK in 2004 [27], ensures that data quality is good for key indicators of health [28–30]. Information from primary care records will be extracted through data entered into structured fields in primary care electronic patient records (Read Codes [31]). Healthcare records contain information on consultation rates, clinical measurement values, prescribing and health-screening [28–30].

### Demographic indicators

Information on patient age, gender and ethnicity will be collected for analyses. Data will be linked to indices for area-level deprivation, such as the index of multiple deprivation [32], at small geographic level.

### Severe mental illnesses

General practitioners are financially incentivised to maintain a register of people with severe mental illnesses. Individuals with a diagnosis of schizophrenia, bipolar affective disorder or non-organic psychosis, comprise people on this register [31]. The use of computer-based electronic records to identify patients with severe mental illnesses in primary care has previously been validated, with a sensitivity of 91 % and positive predictive value of 91 % for non-organic psychosis, assessed against a syndrome checklist derived from the Present State Examination and *International Classification of Disease*-*9* (ICD-9) [33], applied to clinical case notes [34]. Recent work has shown that these diagnostic groupings remain stable over time [35].

### Cardiovascular disease indicators

Diagnostic read codes will be used to ascertain presence of main cardiovascular health indicators. Diabetes mellitus, hypertension, weight (body mass index), smoking status and presence of hyperlipidaemia will be ascertained by presence of diagnostic codes, blood test results and other measurements (e.g., glycated haemoglobin (HbA1c), lipid profile readings, etc.)

### Psychotropic medications

Information on prescriptions of antipsychotic medications according to formulation (oral/depot) and dose, will be extracted and classified [36] prior to analyses.

### Overview of data from secondary care

#### South London and Maudsley NHS Foundation Trust Biomedical Research Centre (SLaM BRC) Case Register

This is an anonymised data resource drawn from the electronic health records of over 250,000 service users who have received care from a large secondary mental health service provider organisation (South London and Maudsley NHS Foundation Trust)37. South London and Maudsley is one of the largest mental health Trusts in Europe serving a base population of approximately 1.2 million people [37] living in south east London and using electronic health records across all its services since 2006 (with some services adopting these earlier). This data source contains all patient contacts with Trust services including out-patient appointments and in-patient admissions. The information from the electronic health record is accessed via the Clinical Record Interactive Search (CRIS) software system, which permits free text and structured fields to be searched for relevant information regarding patient characteristics, interventions and outcomes. All data since 2007 will be used in the E-CHASM study.

### Measures from secondary care

#### Demographic indicators

Information on age, gender and self-ascribed ethnicity will be used. Area-level deprivation indicators matched to the last census will be used in initial analyses; however, work over the course of this study will lead to the derivation of a variable for individual-level socioeconomic position, as detailed below.

### Psychotropic medications

Detail on antipsychotic medications will be derived from the health records and classified into formulation (oral/depot) and dose [36]. Information on medications prescribed will be extracted from structured fields. In order to minimise missing data on prescribed medications, natural language processing with a bespoke algorithm will also be used to extract relevant information on prescribing from free text [38].

### Mental disorder

Within South London and Maudsley Trust (SLaM), clinical teams are required to assign mental disorder diagnoses for all service users [37]. Diagnoses are entered into structured fields on the electronic record. Due to auditing of diagnosis, completion rates for diagnostic fields are high [37]. Using information from these fields, supplemented by natural language processing of free text and clinical note fields [38], the following ICD-10 diagnostic groups will be included in analyses: schizophreniform disorders (F20–F29), mania and bipolar affective disorder (F30, F31).

### Cause-specific mortality

All electronic patient records contained within this dataset have a unique NHS patient identifier which can be linked to death certificate information nationally. Lists of deceased patients are downloaded on a monthly basis from the NHS care records service. To ascertain cause of death, information from linked death certificates will be extracted and categorised according to ICD-10 [39].

### Development of a measure for individual-level socioeconomic position

Much important information is captured within the free-text information fields of electronic patient records [40, 41], particularly in mental healthcare. Using computational techniques such as natural language processing may help to unlock this information from within these text fields [41]. Within the SLaM-BRC Case Register, this has already been applied to derive information on cognition [42], smoking [43], pharmacotherapy [38] and symptoms [44]. The software [General Architecture for Text Engineering (GATE)] is an open source package used for natural language processing [45]. For example, in a study designed to assess smoking use, natural language processing supplemented information within structured fields, leading to an increase in proportions identified as smokers, from 11.6 % (when reliant on structured fields alone) to 64 % (when supplemented by natural language processing of free text) [43]. This approach is robust and repeatable [43]. Algorithms using GATE will be developed to derive indicators for socioeconomic position, through first ascertaining a priori keywords indicative of socioeconomic position, followed by an iterative process of: (1) developing a bespoke gazetteer of terms related to socioeconomic position (together with synonyms), and rules and models for extracting socioeconomic position; (2) evaluating the real world use and applicability of these terms and rules in routine clinical records. At least two indicators of socioeconomic position will be derived: education and occupational social class. Education is a valuable measure of socioeconomic position as it reflects early life socioeconomic position and is strongly related to parental characteristics [46]. Occupational social class is an important measure of socioeconomic position as it taps into individual social standing, conditions relating to work-based stress, and is also predictive of income and material resources [46]. Both have clear associations with mental and physical health [46, 47].

## Analyses

1. To determine ethnic variations in cause-specific mortality patterns among people with severe mental illnesses.

Data from secondary care will be used. ‘Cohorts’ of individuals by severe mental illness diagnoses (e.g., F20 Schizophreniform disorders F30/F31 Bipolar Affective Disorder and Manic episodes) will be followed from 2007 until the latest date at which linked census information on mortality and cause of death are available. Mortality rates by severe mental illness diagnosis will be obtained, indirectly age- and gender-standardised to the population of England and Wales. Indirectly standardised SMRs by ethnic group will also be obtained.

2. To determine ethnic variations in the effect of SMI on cardiovascular risk factors.

Data from primary care will be used. The association of severe mental illness with cardiovascular risk factors (such as type 2 diabetes mellitus, hypertension, obesity, tobacco use and 10-year cardiovascular mortality risk, such as QRISK [48]) will be assessed using multivariable logistic regression, adjusting or stratifying for age and gender as appropriate. Odds ratios stratified by ethnicity for the association of severe mental illness with cardiovascular disease risk will be obtained, in order to assess for ethnic variations in the effect of severe mental illness on cardiovascular risk factors. Formal tests of statistical interaction will be used to assess for heterogeneity in the association of severe mental illness with cardiovascular disease risk factors across strata. The role of putative mediators, such as health-related behaviours, e.g., smoking, antipsychotic medication prescriptions and weight or body mass index, will be assessed using formal procedures to test for mediation [49].

3. To determine ethnic variations in quality of care received, relevant to premature mortality.

‘Poor quality’ of care will be deemed present when therapeutic interventions fall outside of nationally recognised guidelines, in the UK. This might include a failure to adequately screen and manage physical health comorbidities (as detected in primary care data sources), or in the prescribing of antipsychotic medications in excess of recommended dose. Multivariable logistic regression will assess the association of ethnicity (with ‘white British’ as the reference) with each of the care indicators, adjusting or stratifying by age and gender, as appropriate.

4. Validation of the measure for individual-level socioeconomic position, derived using structured field information and natural language processing.

The construct and concurrent validity of the GATE-derived socioeconomic position indicators of education and occupational social class will be assessed using structural equation modelling against a nested cohort of 558 individuals with psychosis [50]. These individuals presented to South London and Maudsley Trust services with a first episode of psychosis between 1st May 2010 and 30th April 2012. Individuals within this cohort were aged 18–64. Information on education and occupation were extracted from patient records by research workers, using the Medical Research Council (MRC) socio-demographic schedule [51].

### Sample size calculation

The following table details detectable effect sizes at 80 % power. There is greater power to detect associations in the primary care sample for each of the ethnic groups, as it is a larger sample (Table 1).

**Table 1:** Smallest effect sizes (odds ratios) detectable for the largest and smallest ethnic minority groups at 80 % power (with two sided 5 % significance levels), for exposures with a prevalence of 10, 20 and 50 % in the reference (white British) group

Primary care data source	*N*	Prevalence of outcome in white British group		
White British	238,211	10 %	20 %	50 %
Smallest detectable odds ratio				
Bangladeshi	93,143	1.03	1.02	1.01
Irish	13,459	1.08	1.05	1.03

Secondary care data source	*N*	Prevalence of outcome in white British group		
white British	28,618	10 %	20 %	50 %
Smallest detectable odds ratio				
‘Other’ white	4477	1.14	1.09	1.05
Indian	711	1.35	1.23	1.11

### Qualitative study

#### Methodology

To address the objectives for the qualitative work, focus groups and interviews will be conducted with patients and clinicians. Focus groups stimulate discussion and involve group processes that can help people to explore and clarify views and provide insight into cultural values and norms. Up to eight focus groups, each comprising 6–8 participants will be facilitated by two interviewers, with one group comprising clinicians and at least one group containing individuals who identify their ethnicity as ‘white British’. In practice, the actual number of focus groups to be conducted will be determined by the range of relevant characteristics that emerge from the quantitative work and how these might best be reflected and balanced within the focus groups [52]. The purpose of the groups will be to understand the perspectives of service users and their carers, in particular experiences of living with cardiovascular disease co-morbid with severe mental illnesses and to identify barriers as well as facilitators to accessing healthcare.

Individual qualitative interviews will then be conducted to further explore the personal experience and relevance of themes identified from the focus groups. It is envisaged that up to twenty individual interviews will be conducted for this purpose.

Purposive sampling will be used to identify participants for both the focus groups and the individual interviews. The criteria for this will be determined after examination of the quantitative data (which may for example give an indication of which ethnic minority groups with severe mental illnesses experience physical health inequalities). This process is described in more detail next.

### Sampling frame for qualitative research

For people living with severe mental illness, the ‘Consent for Contact’ programme [47], a register of South London and Maudsley Trust service users who are willing to be contacted about research projects on the basis of information in their record, will be used to purposively sample participants who have a severe mental illness co-morbid with a known physical health condition. To date, of a total of 9564 service users approached to take part in the ‘Consent for Contact’ programme, 72 % have consented to being contacted for mental health research. There are approximately 1340 individuals with severe mental illnesses, who will form the main pool of participants to be approached to take part in focus groups and interviews for the qualitative part of the study. As this register is linked to the electronic patient record data source detailed above, purposive sampling will be based on characteristics considered important for the composition of the groups (e.g., ethnicity and/or presence of a physical health problem). The composition of focus groups will be informed by findings from the quantitative analyses, which will highlight where inequalities with cardiovascular health in people living with severe mental illness are most pronounced. Stratification [52] will be used to ensure a diversity of people representative of the population are included in the study, dependent on the types of question generated by quantitative findings and may for example include people living with severe mental illnesses who have also been diagnosed with type 2 diabetes mellitus.

### Topic guide for qualitative study

Topic guides will be semi-structured. Domains to be covered in the topic guide will include: experiences of accessing/using mental and physical healthcare, the use of alternative therapies and medical models, experiences of physical symptoms, use of biomedical treatments and disease monitoring, perceptions of stigma/discrimination from health service providers and individuals. Other domains will include perceived barriers to care, including language, recent migration and knowledge of local services. The topic guide will be further developed following feedback from service user representatives.

### Analysis of qualitative data and integration with quantitative findings

Thematic content analysis will be used to identify salient themes, until no further themes emerge. Analyses will be iterative as emergent themes will be used to generate hypotheses which may be tested in the quantitative dataset. Coding of qualitative data will be through appropriate software (N-Vivo) [53]. Emergent themes and coding frameworks will be cross-checked with researchers with expertise in qualitative research. Findings from the qualitative phase will be triangulated with those from the quantitative phase. A triangulation protocol will be used to identify ‘meta-themes’ relevant to findings from both quantitative and qualitative phases of the study [54] and in particular, areas of convergence or divergence.

### Ethical standards

All data will be anonymised and managed according to UK National Health Service (NHS) information governance (IG) requirements.

Ethical approval to examine data from the South London and Maudsley Trust Biomedical Research Centre Case Registry (SLaM BRC case registry) as an anonymised dataset for secondary analysis has been obtained from Oxfordshire REC C in 2008 and renewed in 2013 (reference number 08/H0606/71+5). Methods to de-identify data have been published and are robust [55]. Separate approvals to conduct the analyses proposed within this protocol have been granted by the CRIS Oversight Committee.

The South London Primary Care Research Governance Team reviewed the process of anonymised data analysis for patient data from Lambeth and approved the usage of aggregated anonymised patient data for research purposes. Lambeth Clinical Commissioning Group, Information Governance Steering Group (Lambeth CCG IGCG) has to approve each research project based on individual applications using a standardised proforma, the ‘Privacy Impact Statement’.

For data from east London (Newham, Tower Hamlet, City and Hackney), each of the practices opted into the study by signing forms permitting the Clinical Effectiveness Group (CEG) to use anonymised aggregated data for audits and research projects supported by the CEG. Information Technology (IT) information governance committees provided approval for each of the three localities covered by the CEG.

*Qualitative study* Separate ethical approval will be sought for the qualitative part of the study once the topic guide and likely composition of focus groups have been finalised, following completion of initial analyses of quantitative data.

## Results

### Primary care

The primary care database comprises approximately 1.06 million patient records, including 358,614 anonymised electronic patient records registered to 47 (of 48) general practices in Lambeth and 697,600 anonymised records of patients registered to 142 (of 144) general practices in East London (Tower Hamlets, Newham, City and Hackney) (in total 98.3 % of practices). Data on age and gender are near complete, since these are recorded routinely at patient registration. Self-ascribed ethnicity according to *Office for National Statistics* (ONS) census categories is available for 80–90 % of patients, following local schemes to improve the recording of ethnicity [28–30, 56].

**Fig. 1:**
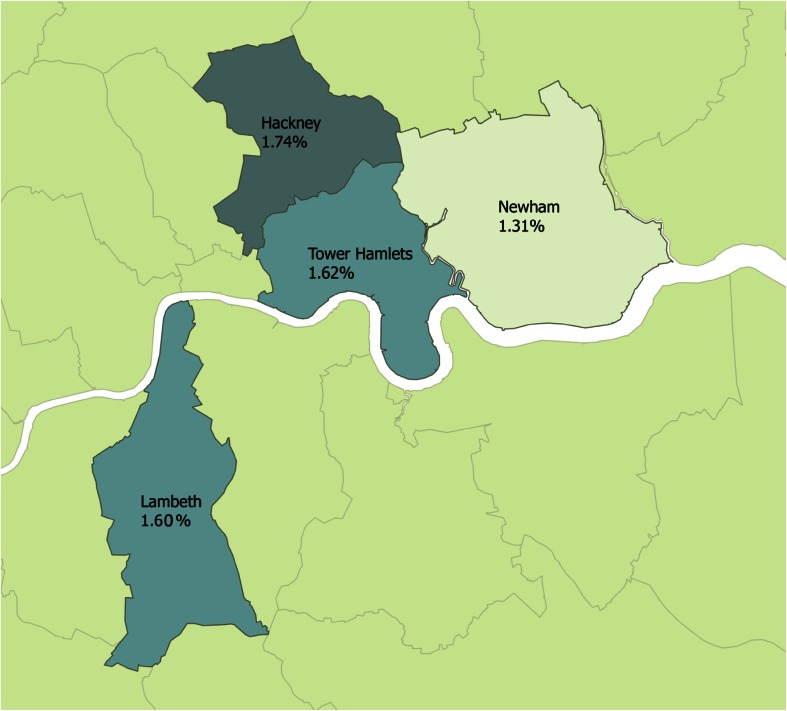
Prevalence of severe mental illnesses across the study sites, by borough

People registered to general practitioners/family doctors within the primary care database are more likely to reside in areas which are deprived and a high proportion of residents in each of the boroughs report their ethnicity as being of minority status (Table 2). Prevalence of severe mental illnesses ranges from 1.3–1.7 %, by borough (Table 2). Figure 1 highlights the geographical distribution of severe mental illness across the study sites.

**Table 2:** Characteristics of primary care database, sample restricted to 18+

	Lambeth	Tower Hamlets	Newham	City and Hackney
Participants N (%)	295,516 (30)	214,600 (21.6)	282,512 (28.4)	200,488 (20.2)
Practices *N* (%)	47 (25)	37 (20)	64 (34)	41 (22)
Proportion resident in most deprived areas^a^ *N* (%)	254,593 (86.2)	169,036 (90.0)	263,681 (98.8)	176,463 (97.7)
Proportion ethnic minorities *N* (%)	152,307 (62.1)	133,727 (68.2)	220,549 (84.0)	118,637 (66.9)
Prevalence severe mental illness				
*N*	4718	3477	3706	3484
% (95 % CI)	1.60 (1.55, 1.64)	1.62 (1.57, 1.67)	1.31 (1.27, 1.35)	1.74 (1.68, 1.80)
Prevalence type 2 diabetes				
*N*	13,372	13,479	20,309	10,754
% (95 % CI)	4.54 (4.47, 4.62)	6.30 (6.20, 6.40)	7.20 (7.11, 7.30)	5.38 (5.28, 5.48)
Prevalence hypertension				
*N*	32,454	22,238	38,632	24,993
% (95 % CI)	10.98 (10.87, 11.10)	10.36 (10.23, 10.49)	13.67 (13.55, 13.80)	12.47 (12.32, 12.61)
Prevalence ischaemic heart disease				
*N*	5103	4792	6759	4226
% (95 % CI)	1.73 (1.68, 1.77)	2.23 (2.17, 2.30)	2.39 (2.34, 2.45)	2.11 (2.05, 2.17)
Current or ex-smoker				
*N*	132,741	48,266	47,749	46,412
% (95 % CI)	46.62 (46.44, 46.80)	50.40 (50.08, 50.72)	40.31 (40.02, 40.59)	50.33 (50.00, 50.65)

^a^Bottom two quintiles for index of multiple deprivation 2000 at lower super output level; prevalence estimates based on number of patients on quality and outcomes framework (QoF) registers for each disease, crude estimates

Compared to people registered to general practitioners and not known to have a severe mental illness, the severe mental illness sample in primary care were older, with a higher proportion of men, a larger proportion of people reporting their ethnicity as white British or black Caribbean and were more likely to reside in deprived areas (Table 3).

**Table 3:** Characteristics of people living with severe mental illnesses in primary care database, sample restricted to adults aged 18+

	Not on SMI register^a^ *N* = 997,731		Severe mental illness (SMI register)^a^ *N* = 15,385		Total
	***N***	%	***N***	%	***N***
Age (mean, SD)	40 (15.5)		47 (15.3)		993,116
Sex					
Men	494,304	51	8488	55	502,792
Women	483,426	49	6897	45	490,323
Ethnicity					
White British	238,211	27	4403	31	242,614
Irish	13,459	2	286	2	13,745
‘Other’ white	171,493	20	1442	10	172,935
Indian	60,298	7	566	4	60,864
Pakistani	35,215	4	381	3	35,596
Bangladeshi	93,143	11	1500	10	94,643
Black Caribbean	43,367	5	1646	11	45,013
Black African	74,037	9	1417	10	75,454
‘Other’/Chinese	110,094	13	2027	14	112,121
Mixed ethnicity	27,877	3	717	5	28,594
Area-level deprivation (quintiles)					
1 Most deprived	587,820	64	10,437	71	589,257
2	261,966	29	3550	24	265,516
3	51,207	6	521	4	51,728
4	10,396	1.1	96	0.7	10,492
5 Least deprived	4626	0.5	21	0.1	4647

^a^Severe mental illness refers to patients with any of schizophrenia, schizoaffective disorder, bipolar affective disorder and any non-organic psychosis

*p* < 0.001 for all sociodemographic variables; comparing SMI to non-SMI group

### Secondary care

The secondary care database currently comprises approximately 260,000 anonymised patient records [37], and has increased consistently by around 20,000 per year. Age and gender are complete in this database. Ethnicity is self-ascribed according to standardised criteria, consistent with the last census. Information on self-ascribed ethnicity was 93–97 % complete in 2007–2013. Table 4 displays ICD-10 diagnoses for severe mental illnesses by ethnicity, for this database.

**Table 4:** Breakdown of severe mental illness diagnosis by ethnicity in secondary care database, 2007–2013

	Schizophrenia	Schizoaffective disorder	Bipolar affective disorder
	*N* = 6885	*N* = 1438	*N* = 4112
Ethnicity, *n* (%)			
White British	2271 (33)	519 (36)	2033 (49)
‘Other’ white	473 (7)	93 (6)	376 (9)
Irish	141 (2)	37(3)	132 (3)
Indian	131 (2)	21 (1)	64 (2)
Pakistani	55 (0.1)	10 (0.7)	24 (0.6)
Bangladeshi	29 (0.4)	8 (0.6)	18 (0.4)
Black Caribbean	1004 (15)	173 (12)	255 (6)
Black African	926 (13)	194 (13)	273 (7)
‘Other’/Chinese	1472 (21)	284 (20)	606 (15)
Mixed ethnicity	97 (1)	22 (1.5)	48 (1)
Not stated/missing	286 (4)	71 (4.9)	257 (6)

## Discussion

People living with severe mental illnesses experience a dramatic reduction in life expectancy [1], a large proportion is accounted for through cardiovascular disease [3]. Although complex [57], a parallel body of work has highlighted the particular problem of cardiovascular disease for many ethnic minority groups [21, 58, 59]. It is, therefore, surprising that there is a dearth of evidence, relating to cardiovascular disease in ethnic minority groups living with severe mental illnesses [14]. This is a concern, as this represents preventable causes of death. The present study, E-CHASM, will address this gap in knowledge.

The results presented suggest that the prevalence of severe mental illness in the study catchment area is greater than previously published estimates for prevalence of psychotic disorders in Britain, which have been noted to range from 0.4 to 0.8 %, with considerable variability between geographical regions [60]. Local areal estimates for severe mental illness are, however, broadly consistent with those published by Public Health England [59]. Cardiovascular disease indicators presented here are broadly consistent with prevalence estimates published for London [60].

The feasibility of E-CHASM rests on its usage of routine electronic patient records to establish differences in prevalence and treatment access. In particular, analyses of records from patients registered to general practices in an ethnically and socioeconomically diverse region in a major inner-city conurbation, alongside analysis of records from a large mental health Trust serving these populations, will enhance the study. The enriched representation of populations normally under-represented or absent in similar work [14] will allow the assessment of ethnic minority physical health inequalities in severe mental illness populations. Methodological techniques such as natural language processing to data-mine free text within secondary care records [41, 45] will enable development of measures for socioeconomic position through robust and repeatable methods which will also enable the automation of checking of a large volume of records which would otherwise be impossible [41]. As far as we are aware, the derivation of an individual-level measure for socioeconomic position from routine electronic health records has not been previously attempted. Finally a novel application of using electronic patient records in research is in the application of the ‘consent for contact’ programme at South London and Maudsley Trust. Thus, following analysis of quantitative data, it will be possible to purposively sample potential participants who will be invited to take part in focus groups and individual interviews for the qualitative phase of this study, based on important attributes (e.g., type of diagnosis, ethnicity, presence of physical comorbidity). Thus, the findings from the quantitative phase will directly inform qualitative data collection. This form of integration, known as ‘connecting’, will draw from the strengths of deductive methods in the quantitative phase to inform study design for the qualitative phase [22]. Integration of findings across qualitative and quantitative data sources [54] will help to understand mechanisms underlying quantitative findings as well as identify barriers to care from the perspectives of service users, carer and service providers. A future application of this data source could be to assess discrepancies in care provided across primary and secondary care. This would be based on data linkages between primary and secondary care, which could be explored in future work.

## Strength and limitations

Strengths of this study include the power to conduct statistical analyses in groups of individuals who form a minority in the population and thus address the current scarcity of research in this field. Other strengths include the usage of natural language processing to derive a measure for individual-level socioeconomic position. If successful, this will provide a methodological advantage, as almost all previous work using electronic health records has tended to rely on area-level measures for deprivation. The mixed methods design of the study will enhance possibilities of understanding trends in quantitative analyses as well as highlighting barriers to equitable care from the perspectives’ of service users and service providers. In all of the quantitative data sources, ethnicity is self-ascribed. This is an addition over previous work which has tended to rely on country of birth [61].

Limitations relate to analysing routine electronic patient records, where missing data may be associated with bias and loss of precision [62]. There may also be concerns around the quality of the data entered on databases and variables to adjust for known confounders may not be available [63]. It may be possible to apply specialist techniques to address this [62–64]. For the cross-sectional phases of the study, it will not be possible to conclude temporality of associations.

## Dissemination

Analyses will be disseminated in peer-reviewed manuscripts and through conference proceedings. If requested, analyses will also be prepared as reports or presentations for interested stakeholders. Findings relating directly to clinical care will be fed back to clinical care networks, with a view to informing guideline development.

## Conclusions

There is currently an absence of evidence relating to life expectancy differences in ethnic minority people living with severe mental illnesses such as schizophrenia. In particular, little is known about the experience of cardiovascular disease in these populations and whether there are additional barriers or inequities in service provision. E-CHASM will seek to address these gaps in knowledge through a combination of quantitative analysis of electronic health records and qualitative interviews.
